# Pilot Investigation: Testing Opaque Water as an Agonism Mitigating Visual Barrier for Nile Crocodiles (*Crocodylus niloticus*)

**DOI:** 10.1002/zoo.70022

**Published:** 2025-09-03

**Authors:** Austin Leeds, Andy Daneault, Alex Riley, Laura Stalter, Kristen Wolfe, Ike Leonard, Andrew C. Alba, Joseph Soltis

**Affiliations:** ^1^ Disney's Animal Kingdom® Lake Buena Vista Florida USA

**Keywords:** agonism, animal welfare, crocodilian, exhibit complexity, visual barrier

## Abstract

This pilot investigation sought to evaluate the effectiveness of water opacity as an agonism‐mitigation strategy in an all‐male group of Nile crocodiles (*Crocodylus niloticus*). Crocodile behavior was monitored over 95 observation hours, split approximately equally between transparent water and opaque water conditions. In addition to agonism rates, the crocodiles' use of water was evaluated to ensure the change in their habitat did not disrupt utilization of the water, which is an important component of their thermoregulatory processes. Agonism rates were approximately equal between the transparent and opaque water conditions. The proportion of the group in water was lower in the opaque condition though the mean difference between conditions suggests that approximately two more crocodiles (out of a group size that ranged from 21 to 18 individuals) were in water in the transparent condition than opaque condition. Overall, these findings suggest the opaque water had little to no effect on the crocodiles' behavior as measured here. However, the opaque water may have increased environmental complexity. In nature, crocodiles navigate opaque water utilizing chemosensory and integumentary sensory modalities that are likely underutilized in transparent water commonly provided to crocodilians living in human care. Further research is needed to understand how opaque water may be enriching for crocodilians.

## Summary

- Water opacity had no effect on agonism in this group of Nile crocodiles.
- The crocodiles' utilization of water was slightly reduced in the opaque water condition. This avoidance may have been a neophobic response to a novel change in their environment.
- Crocodilians utilize uniquely evolved sensory modalities to navigate opaque water in nature. Manipulating water opacity as a means for crocodilians to utilize these sensory capabilities in human care should be studied as a potential enrichment/environmental complexity component of their husbandry.

## Graphical abstract

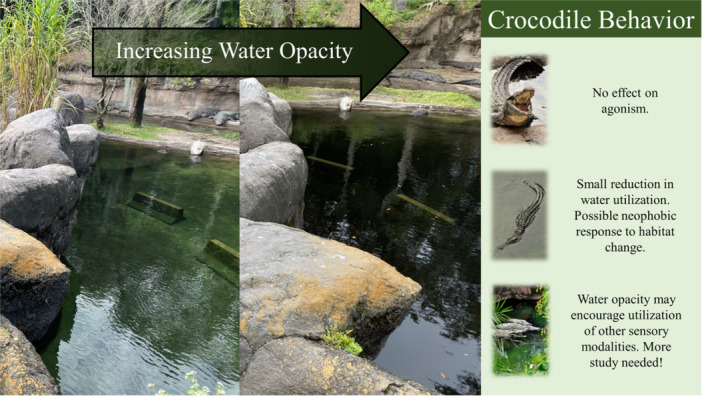

## 1 Introduction

Agonism is a species‐typical behavior utilized to manage social relationships (e.g., Wheeler et al. 2013); however, agonism can contribute negatively to welfare statuses (Salas et al. 2016). Thus, managers of animals in human care have a responsibility to mitigate agonism that may negatively affect an animal's welfare status. Ad hoc placement of visual barriers within animal living spaces is an easily implemented low‐cost approach to mitigating agonism. Visual barriers are thought to reduce sight lines between conspecifics and/or create escape routes, ultimately reducing the incidence of agonism. Though commonly discussed as an agonism mitigation strategy, only a few case studies have demonstrated their effectiveness (Deeming et al. 2011; Erwin et al. 1976; Hasegawa and Maekawa 2009; Valuska et al. 2013; Waran and Broom 1993).

At Disney's Animal Kingdom®, we care for a group of Nile crocodiles (*Crocodylus niloticus*). During the winter, the species' breeding season, we observe increases in agonism (Leeds et al. 2024). Mitigating agonism during this period has been a priority for which we have explored the use of visual barriers. Nearly all the crocodiles' agonism occurs in water (Leeds et al. 2022). Thus, our first visual barrier test utilized logs that rested at the water surface in locations where agonism was most frequent. We hypothesized this would break up sight lines while crocodiles were in their species‐typical minimal exposure posture (i.e., eyes, nostrils, ears, cranial platform above water surface, rest of body below; Nagloo et al. 2016). Both short‐ and long‐term monitoring found this to be ineffective at reducing agonism (Leeds et al. 2022).

In nature, Nile crocodiles live in rivers, estuaries, and lakes that have varying intensities of water opaqueness. However, the crocodiles at Disney's Animal Kingdom®, like many zoo‐living crocodilians, live in transparent water to facilitate guest viewing. While visual barriers are typically physical structures (e.g., Brien et al. 2014; Meulendijks et al. 2024; Valuska et al. 2013), manipulating water opacity could be utilized as a form of visual barrier. To the author's knowledge, this approach has not been evaluated in zoological institutions, but laboratory studies have found agonism in various fish species to both decrease (Zulfahmi et al. 2024) and increase (Gray et al. 2012) with increasing water opaqueness. Here we evaluated the effect of increasing water opacity on agonism rates within this group of crocodiles. We hypothesized that increasing water opacity would reduce agonism by decreasing the visibility of conspecifics. Our previous visual barrier study found that the crocodiles initially avoided the water when barriers were placed in the exhibit (Leeds et al. 2022). As time spent in water may affect thermoregulation, we also monitored time in water to ensure no negative changes occurred.

## 2 Methods

### 2.1 Subjects and Water Opacity

An all‐male group of Nile crocodiles living at Disney's Animal Kingdom®, Lake Buena Vista, FL, USA, were observed. Group size was 21 and 18 adult individuals during the transparent and opaque water conditions, respectively. The group lived in an outdoor habitat that included beaches, islands, and a large water body that was viewed by guests via a safari truck ride. Floating logs from the previous visual barrier study were present in both study conditions (Leeds et al. 2022).

To increase water opacity, Black Out Ultra Concentrate (Sanco Industries, Inc.; Fort Wayne, IN) was added to a water supply line via a meter pump. The meter pump distributed each dose evenly over the specified period. As a loading dose, 0.315 L of the solution were administered over 12 min on both Day 1 and 2 of the opaque condition. After the Day 2 loading dose, maintenance dosing began during which 0.19 L were administered over a 24 h period through Day 22. On Day 23, the ozone filtration system, which had been turned off for the previous 22 study days, was brought back online. To maintain water opacity the 24 h dose was raised to 0.76 L on Day 23, and to 1.14 L on Day 24 through 29 (end of study). Water opacity was quantified via Secchi disk submersion. Secchi disk visibility was 100% at approximately 5 ft (deepest point of habitat) in transparent water. Secchi disk visibility was 0% at approximately 1 ft in opaque water. Water temperature was controlled by a heating system and was approximately equal between observation conditions (*M*_transparent _= 77.2°F, SD = 0.74; *M*_opaque_ = 77.3°F, SD = 0.81).

### 2.2 Observations

**Figure 1:**
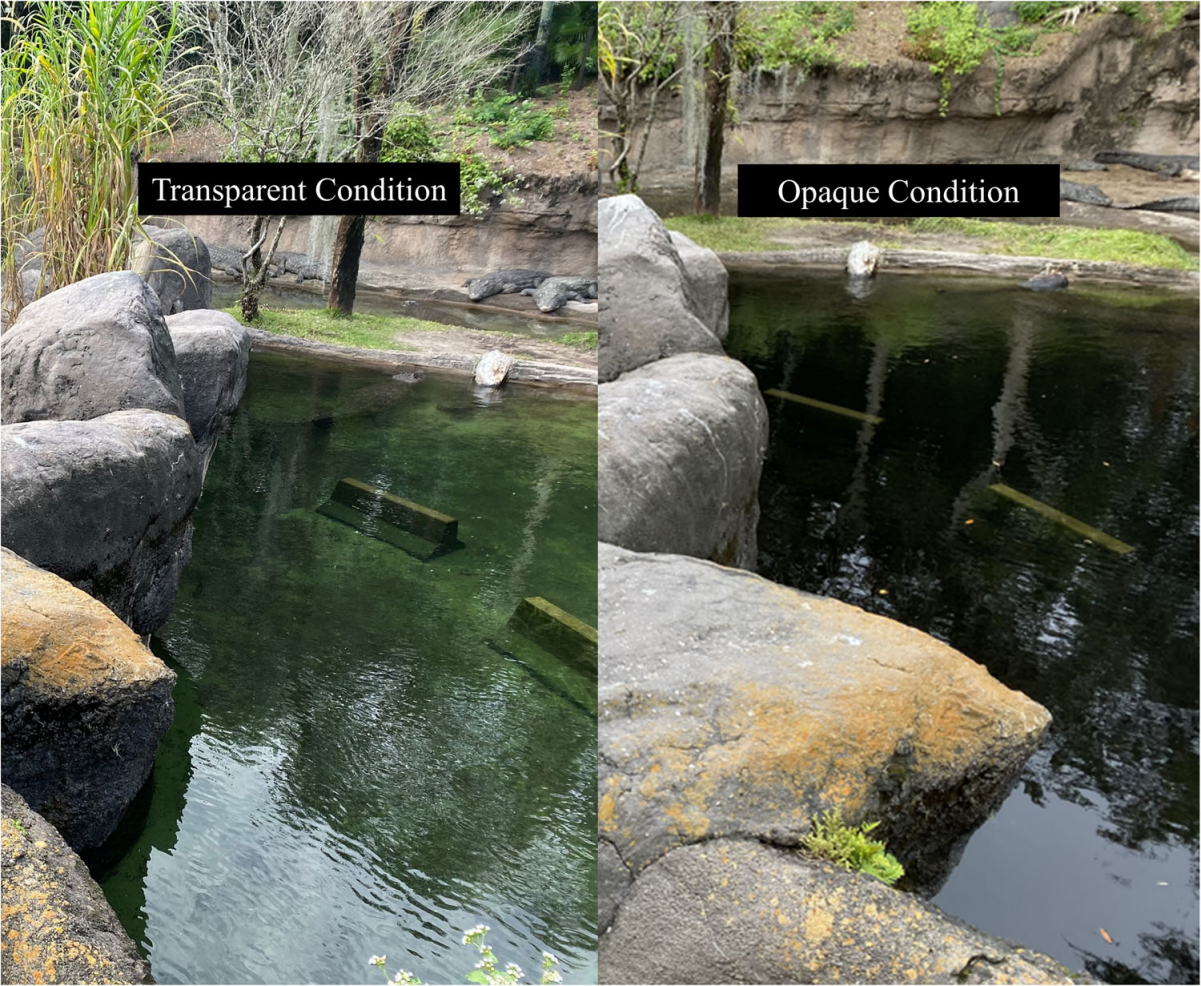
Visual of water transparency from safari vehicle roadway. Left image is of water in the transparent condition. Note the Jersey barriers and bottom of water feature are fully visible. Right image is of water in the opaque condition. Note only the top of the Jersey barrier is visible.

Crocodile behavior was observed in two water conditions: transparent and opaque (Figure 1). The transparent condition occurred January 27, 2021–February 27, 2021 (*n*_observations _= 46). The opaque condition occurred January 27, 2022–February 27, 2022 (*n*_observations _= 49). This match‐control methodology of comparing the same months a year apart was used to account for distinct seasonal differences in the crocodiles' behavior (Leeds et al. 2024). We conducted 60 min observations up to twice daily between 07:00 a.m. and 17:00 p.m. All occurrence sampling of agonistic bouts were recorded and the number of crocodiles in water was recorded at three scans during each hour observation (0, 30, and 60 min). For full observation details see Supporting Information S1.

### 2.3 Analysis

Analysis was conducted in R (v. 4.4.1; R Core Team 2024). Counts of agonistic bouts per observation and the proportion of the group in water per scan were the dependent variables for the generalized linear mixed models (glmmTMB function, Brooks et al. 2017). For agonism, the overall rate, the rate on land, and rate in water were analyzed to evaluate if the water dye reduced agonism overall and/or by location where the dye was (water) and wasn't (land) present. Agonism was modeled with a Poisson distribution and log link function. Water use was modeled with a binomial distribution and logit link function. Condition (transparent/opaque), time of day (morning/midday/afternoon) and air temperature (°F) were included as fixed factors in both models. Time of day and temperature were included as control variables, as previous research has shown both factors influence behavior (Leeds et al. 2022, 2024; Riley et al. 2021). Observation day was included as a random factor in both models to account for multiple observations occurring per day. For the agonism model, group size was included as an offset to correct for differences in group size between conditions. For interpretability of this offset, estimated marginal means (EMM) were scaled to 1 (i.e., value equals mean agonism rate per individual per observation hour). Residual normality was confirmed via QQ plot, and no multicollinearity was detected (vif scores ≤ 1.5).

Dichotomous statistical values are not reported (Amrhein et al. 2019; Berner and Amrhein 2022; Halsey 2019). Instead, modeled effect size measures are presented to describe differences in behavior by condition. First, EMMs with a 95% confidence level (CL) are reported with values rounded to three decimal places. Second, effect size ratios and 95% CL were calculated (emmeans function, Lenth 2024). For agonism, an EMM ratio (EMM_ratio_) was calculated as the quotient of the two contrasted EMM values. For example, an EMM_ratio_ of 1.0 means values are equivalent (numerator = denominator) while a ratio of 2.0 means the numerator was two times greater than the denominator. For proportion of group in water, an odds ratio (OR) was calculated. Numeric values are rounded to three decimal places. Full model outputs and additional data summarization are provided in Supporting Information S2.

### 2.4 Ethical Approval and Conflict of Interest

Study methods were approved by the scientific review committee of Disney's Animal Kingdom®. The authors declare no conflicts of interest.

## 3 Results

**Figure 2:**
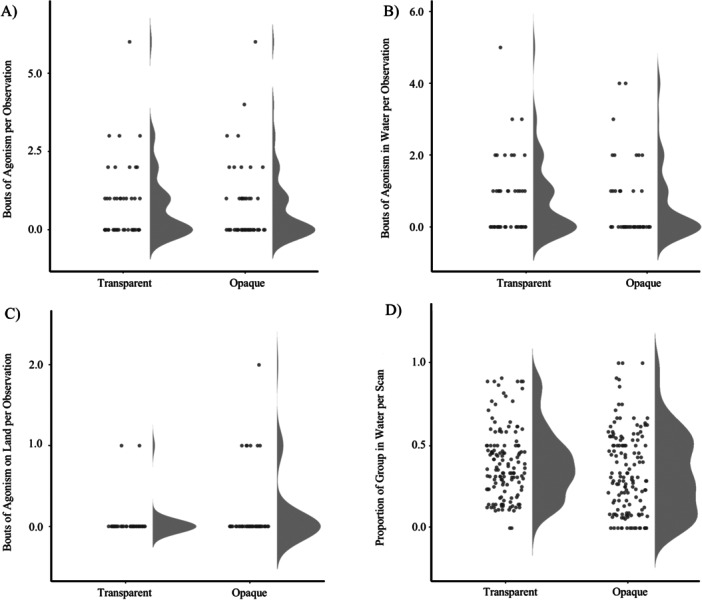
(A) Frequency of agonistic bouts per observation by water condition. (B) Frequency of agonistic bouts in water per observation by water condition. (C) Frequency of agonistic bouts on land per observation by water condition. (D) Proportion of group in water per scan by water condition.

The overall mean rate of agonism was equal between the opaque (EMM = 0.028, CL: 0.017, 0.047) and transparent conditions (EMM = 0.028, CL: 0.017, 0.046; Figure 2A). The EMM_ratio_ was 1.000 (CL: 0.533, 1.890). The mean rate of agonism in water was similar between the opaque (EMM = 0.020, CL: 0.011, 0.037) and transparent conditions (EMM = 0.026, CL: 0.015, 0.043; Figure 2B). The EMM_ratio_ was 1.26 (CL: 0.623, 2.510). The mean rate of agonism on land approached zero in both conditions but was on average larger in the opaque condition (EMM = 0.003, CL: 0.003–0.018) compared to the transparent condition (EMM = 0.001, CL: 0.000–0.007; Figure 2C). The EMM_ratio_ was 3.270 (CL: 0.688, 15.500). The mean proportion of group in water was greater in the transparent condition (EMM = 0.363, CL: 0.289, 0.444) than in the opaque condition (EMM = 0.275, CL: 0.217, 0.342; Figure 2D), though the absolute difference between means was relatively small with broadly overlapping CL's. The OR suggests the odds of being in water was 50% greater in the transparent condition though the CL was wide suggesting uncertainty in the estimation (OR = 1.500, CL: 0.946, 2.380).

## 4 Discussion

While water opacity may have reduced these crocodiles' underwater visual acuity, the opacity likely had a limited effect on their chemoreceptor (Weldon and Ferguson 1993) and integumentary sense organs (Grigg and Kirshner 2015) that are also utilized in the identification of conspecifics. These additional sensory capacities are likely why water opacity did not reduce agonism in this study. On average, rates were higher on land in the opaque condition; however, the rates in both conditions approached zero so it's likely this relatively small difference is the result of sampling variation.

On average, the crocodiles were less likely to be in opaque than transparent water. This may have been a neophobic response to a change in their environment. A similar pattern of behavior was observed when logs were added to their habitat (Leeds et al. 2022). It is worth noting that on average only two more crocodiles were in water at any scan during the transparent condition. Given the size of our group this suggests this difference in behavior may not be meaningful from a practical animal management or welfare perspective. However, we suggest that future studies include a sufficient adjustment period in their methodology as both this and our previous study suggest crocodiles may have a small to moderate neophobic reaction to novel environmental changes.

Providing complex living spaces for animals in human care is an important contributor to their welfare status (de Azevedo et al. 2023). While water opacity had little effect on behavior in this study, it is possible that it provided novel environmental complexity. Crocodilians naturally live in opaque water and have evolved sensory modalities to navigate their environments. Reptile welfare is notably understudied (Binding et al. 2020), and it is unknown how significant living in an environment where these senses are less utilized is to an individual's welfare status. To this end, we continue to maintain the exhibit water opaque for the purpose of increasing exhibit complexity.

Ultimately, there are large gaps in our knowledge of reptilian care and welfare (Mendyk and Warwick 2023), and water opacity may have value in other contexts not evaluated here. For example, water opacity may encourage more exploration and/or swimming within the exhibit if individuals feel less visually exposed to conspecifics. Opacity may also be effective for managing introductions where social interactions may be more intense than what was observed in this group that has lived together for more than 25 years. There is much to evaluate in terms of crocodilian care and welfare, and we hope this investigation encourages further research into the matter.

## Supporting information

Supplementary_Material_Methods.

**Table S1**: Agonism in water model output. **Table S2**: Agonism on land model output. **Table S3**: Space use model output. **Table S4**: Estimated marginal mean (95% confidence level) rates of agonism by time of day (morning, midday, afternoon), water condition (transparent, opaque) and physical location (in water, on land). **Table S5**: Estimated marginal mean (95% confidence level) of the proportion of group in water by time of day (morning, midday, afternoon) and water condition (transparent, opaque).

## Data Availability

The data that support the findings of this study are available from the corresponding author upon reasonable request.
