# Supplementary material for: Pilot Investigation: Testing Opaque Water as an Agonism Mitigating Visual Barrier for Nile Crocodiles (Crocodylus niloticus)
Source: Zoo Biol. 2025 Sep 3;44(6):671–5. doi: 10.1002/zoo.70022 (PMC12681046; doi:10.1002/zoo.70022)
Supplement: Supplementary file 1 — Supplementary_Material_Methods. [file ZOO-44-671-s001.docx]

**Supplementary methodological material for manuscript “Pilot investigation: testing opaque water as an agonism mitigating visual barrier for Nile crocodiles (*Crocodylus niloticus*)”**

***Detailed Observation Methodology***

Crocodile behavior was observed in two water conditions: transparent and opaque. The transparent condition occurred January 27 - February 27, 2021 (n_observations_ = 46). The opaque condition occurred January 27 - February 27, 2022 (n_observations_ = 49). This match-control methodology of comparing the same months a year apart was used to account for distinct seasonal differences in the crocodiles’ behavior (Leeds et al. 2024). Behavior was sampled during one-hour observations conducted up to twice daily between 07:00 and 17:00 (balanced across all hours over the course of study; Table S1) via a remote camera system. The camera system provided greater exhibit visibility (>90%) than if conducting observations in person and removed observer presence as a potential confounding variable. These observation times were selected as they provided the most consistent day light hours to observe the crocodiles. The camera system was not effective at consistently observing behavior when sunlight was unavailable and thus these observation hours were not included.

**Table S1**. Count of observations by hour and study condition.

|  | **Observations** | |
| --- | --- | --- |
| **Observation Hour** | **Transparent Condition** | **Opaque Condition** |
| **7:00** | 5 | 5 |
| **8:00** | 6 | 6 |
| **9:00** | 3 | 3 |
| **10:00** | 5 | 6 |
| **11:00** | 4 | 4 |
| **12:00** | 4 | 4 |
| **13:00** | 3 | 4 |
| **14:00** | 5 | 6 |
| **15:00** | 6 | 6 |
| **16:00** | 5 | 5 |
| **Total** | 46 | 49 |

During observations the crocodiles’ social interactions and space use patterns were documented. Social interactions were recorded via all-occurrence sampling. Social interactions were categorized as agonistic bouts. Agonistic bouts were defined as aggressive or intolerant behaviors, directed from one crocodile toward a conspecific, resulting in physical contact (Brien et al., 2014), including jaw clashes and bites. A jaw clash was defined as two crocodiles striking heads together with their mouths. A bite was defined as one crocodile closing one’s jaws around a conspecific, possibly including a hold (>1s in which jaws remain closed on conspecific).

Space use data were collected via scan sampling three times per 60 min observation (0 min, 30 min, 60 min). Space use was used to assess the number of crocodiles in water, defined as an individual with >50% of their body in water. Data were collected by AL, AR, LS, and ACA. Interobserver reliability was established amongst all collectors by coding video previously reviewed by AL and AR. Reliability was established if 80% agreement for each behavioral measure was achieved. All differences in coding were reviewed and discussed even if reaching the 80% minimum agreement. If reliability was not established, additional practice videos were provided, and an observer was then tested again. Once reliability was achieved, an observer could conduct observations that contributed to the dataset. Some interactions could be challenging to code due to a variety of factors including glare and splashing. If such an event occurred during a study observation our methods encouraged sharing these events with all observers for discussion and consensus coding. This approach provided additional sporadic training for the team throughout the course of study.

In addition to the described behaviors, abiotic factors were documented for each observation. Abiotic factors were time of day (morning, 07:00­­–10:00; midday, 11:00­–14:00; afternoon, 15:00–­18:00), temperature (°F at end of observation hour).
