# Supplementary material for: Pilot Investigation: Testing Opaque Water as an Agonism Mitigating Visual Barrier for Nile Crocodiles (Crocodylus niloticus)
Source: Zoo Biol. 2025 Sep 3;44(6):671–5. doi: 10.1002/zoo.70022 (PMC12681046; doi:10.1002/zoo.70022)
Supplement: Supplementary file 2 — Table S1: Agonism in water model output. Table S2: Agonism on land model output. Table S3: Space use model output. Table S4: Estimated marginal mean (95% confidence level) rates of agonism by time of day (morning, midday, afternoon), water condition (transparent, opaque) and physical location (in water, on land). Table S5: Estimated marginal mean (95% confidence level) of the proportion of group in water by time of day (morning, midday, afternoon) and water condition (transparent, opaque). [file ZOO-44-671-s002.docx]

**Supplementary results material for manuscript “Pilot investigation: testing opaque water as an agonism mitigating visual barrier for Nile crocodiles (*Crocodylus niloticus*)”**

**Table S1**. Agonism in water model output.

| Model Factor | Factor Category | Estimate | Standard Error | Z Value |
| --- | --- | --- | --- | --- |
| Intercept | - | -2.249 | 1.365 | -1.648 |
| Condition | Transparent | 0.230 | 0.352 | 0.654 |
|  | Opaque | - | - | - |
| Time of Day | Morning | 0.349 | 0.410 | 0.852 |
|  | Midday | -0.114 | 0.452 | -0.252 |
|  | Afternoon | - | - | - |
| Temperature | - | -0.025 | 0.018 | -1.421 |

**Table S2**. Agonism on land model output.

| Model Factor | Factor Category | Estimate | Standard Error | Z Value |
| --- | --- | --- | --- | --- |
| Intercept | - | -6.539 | 2.816 | -2.322 |
| Condition | Transparent | -1.492 | 0.795 | -1.877 |
|  | Opaque | - | - | - |
| Time of Day | Morning | 1.563 | 1.214 | 1.288 |
|  | Midday | 1.803 | 1.152 | 1.565 |
|  | Afternoon | - | - | - |
| Temperature | - | 0.008 | 0.035 | 0.226 |

**Table S3**. Space use model output.

| Model Factor | Factor Category | Estimate | Standard Error | Z Value |
| --- | --- | --- | --- | --- |
| Intercept | - | 1.747 | 0.816 | 2.140 |
| Condition | Transparent | 0.405 | 0.235 | 1.723 |
|  | Opaque | - | - | - |
| Time of Day | Morning | 0.307 | 0.176 | 1.743 |
|  | Midday | -0.364 | 0.159 | -2.295 |
|  | Afternoon | - | - | - |
| Temperature | - | -0.0399 | 0.011 | -3.743 |

**Table S4**. Estimated marginal mean (95% confidence level) rates of agonism by time of day (morning, midday, afternoon), water condition (transparent, opaque) and physical location (in water, on land). Values less than 0.001 are rounded to 0.000.

|  | In Water | | On Land | |
| --- | --- | --- | --- | --- |
|  | Transparent | Opaque | Transparent | Opaque |
| Morning | 0.034  (0.018, 0.063) | 0.028  (0.013, 0.054) | 0.002  (0.000, 0.012) | 0.012  (0.000, 0.033) |
| Midday | 0.021  (0.010, 0.046) | 0.017  (0.007, 0.039) | 0.003  (0.000, 0.016) | 0.015  (0.000, 0.016) |
| Afternoon | 0.024  (0.012, 0.048) | 0.019  (0.009, 0.040) | 0.000 (0.000, 0.006) | 0.002  (0.000, 0.019) |

**Table S5**. Estimated marginal mean (95% confidence level) of the proportion of group in water by time of day (morning, midday, afternoon) and water condition (transparent, opaque).

|  | Transparent | Opaque |
| --- | --- | --- |
| Morning | 0.441  (0.353, 0.533) | 0.345  (0.269, 0.429) |
| Midday | 0.287  (0.216, 0.371) | 0.212  (0.158, 0.279) |
| Afternoon | 0.367  (0.281, 0.463) | 0.279  (0.211, 0.359) |
